# HNRNPA1-mediated exosomal sorting of miR-483-5p out of renal tubular epithelial cells promotes the progression of diabetic nephropathy-induced renal interstitial fibrosis

**DOI:** 10.1038/s41419-021-03460-x

**Published:** 2021-03-10

**Authors:** DongWei Liu, FengXun Liu, ZhengYong Li, ShaoKang Pan, JunWei Xie, ZiHao Zhao, ZhenJie Liu, JiaHui Zhang, ZhangSuo Liu

**Affiliations:** 1 Department of Nephrology, the First Affiliated Hospital of Zhengzhou University, Zhengzhou, 450052 PR China; 2 Research Institute of Nephrology, Zhengzhou University, Zhengzhou, 450052 PR China; 3 Research Center for Kidney Disease, Zhengzhou, Henan 450052 PR China; 4 Key Laboratory of Precision Diagnosis and Treatment for Chronic Kidney Disease in Henan Province, Zhengzhou, 450052 PR China; 5 Core Unit of National Clinical Medical Research Center of Kidney Disease, Zhengzhou, 450052 PR China

**Keywords:** Cell biology, Molecular biology

## Abstract

Diabetic nephropathy (DN) is a serious complication in type 1 and type 2 diabetes, and renal interstitial fibrosis plays a key role in DN progression. Here, we aimed to probe into the role and potential mechanism of miR-483-5p in DN-induced renal interstitial fibrosis. In this study, we corroborated that miR-483-5p expression was lessened in type 1 and type 2 diabetic mice kidney tissues and high glucose (HG)-stimulated tubular epithelial cells (TECs), and raised in the exosomes derived from renal tissues in type 1 and type 2 diabetic mice. miR-483-5p restrained the expressions of fibrosis-related genes in vitro and renal interstitial fibrosis in vivo. Mechanistically, miR-483-5p bound both TIMP2 and MAPK1, and TIMP2 and MAPK1 were bound up with the regulation of miR-483-5p on renal TECs under HG conditions. Importantly, HNRNPA1-mediated exosomal sorting transported cellular miR-483-5p out of TECs into the urine. Our results expounded that HNRNPA1-mediated exosomal sorting transported cellular miR-483-5p out of TECs into the urine, thus lessening the restraint of cellular miR-483-5p on MAPK1 and TIMP2 mRNAs, and ultimately boosting extracellular matrix deposition and the progression of DN-induced renal interstitial fibrosis.

## Introduction

Diabetic nephropathy (DN) is one of the common microvascular complications in type 1 or type 2 diabetic patients, and it can gradually develop into end-stage renal disease (ESRD)^1,2^. The pathogenesis of DN is very complicated, among which renal interstitial fibrosis is a common way for DN to develop into ESRD^3^. The main reason for renal interstitial fibrosis is the excessive accumulation of extracellular matrix (ECM) in the renal interstitial^4^. Therefore, further elucidation of the underlying molecular mechanism of renal interstitial fibrosis in DN is conducive to relieve DN.

MicroRNAs (miRNAs) are small and endogenous non-coding RNAs, and negatively regulate the expressions of their target genes by binding to the 3′ untranslated region (UTR) of mRNAs^5,6^. Importantly, increasing evidence expounds that the dysregulation of miRNAs is bound up with the regulation of the occurrence and development of renal fibrosis in DN. For instance, previous research demonstrates that in the diabetic db/db mouse model, the reduction of miR-204 boosts albuminuria and cortical fibrosis, hinting that the endogenous miR-204 protects the kidneys from chronic injury by diabetes^7^, and another study expounds that miR-21 is abnormally highly expressed in the kidney tissues of diabetic patients, and the restraint of miR-21 alleviates DN interstitial fibrosis and ameliorates other functional parameters of DN mice^8^. Here, we aimed to probe into a miRNA that worked both in type 1 diabetes and type 2 diabetes, and we corroborated that miR-483-5p (also named miR-483) had abnormal expression in both type I and type II diabetic mice through the different database searches, and this finding was further corroborated by quantitative real-time PCR (qRT-PCR). Therefore, we believed that miR-483-5p was worthy of our further research.

In the current study, we mainly applied streptozotocin (STZ)-induced type 1 diabetic mouse model and db/db type 2 diabetic mouse model and high glucose (HG)-induced renal tubular epithelial cell (TEC) model to probe into the function of miR-483-5p in renal interstitial fibrosis in DN and probed into the possible mechanism of changes in the expression of miR-483-5p in the kidney.

## Materials and methods

### Establishment of diabetic mice model

Male C57BL/6 mice (6–8 weeks) were applied to the construction of type 1 diabetic model. Five mice were randomly assigned to each group. The investigator was blinded to the group allocation.

A mouse model of type 1 diabetes was induced by intraperitoneal injection of 50 mg/kg body weight of STZ (Sigma-Aldrich) in 100 mmol/L sodium citrate (pH 4.5) in mice (*n* = 5) for 5 consecutive days. Moreover, db/db mice (*n* = 5) were applied as the type 2 diabetic model. All animals were housed in cages with controlled temperature and humidity, maintained in a light–dark cycle of 12 h to 12 h, and allowed to adapt to the environment for 2 weeks before the study. Besides, the detection of blood glucose was conducted in diabetic mice and the blood glucose concentration of 11.1 mM on the third day after STZ injection was considered that the successful establishment of diabetic mice model. The blood glucose concentration of the mice was continuously tested at week 4, 8, 12, 16, and 20. All the mice were euthanized at the 20th week, and 24-h urine and kidney tissues were harvested. All animal experiment protocols were approved by the Animal Care and Use Committee of Zhengzhou University.

### Masson staining

The kidney tissues of the mice were fixed and made into slices. For the assessment of the pathological changes of mouse kidneys, we applied Masson’s Trichrome Stain Kit (Solarbio) to conduct the Masson staining experiments given the standard protocol provided by the reagent manufacturer.

### Isolation and identification of exosomes

Given the previously described method with minor changes^9^, we carried out the extraction of the exosomes derived from the renal tissues. Specifically, we gathered the renal cortex (100 mg) and digested the tissues with collagenase and trypsin at room temperature for nearly 2 h. Then, the samples were centrifuged at 1500 × *g* for 25 min to remove cells and residual debris, followed by gradient centrifugation (13,000 *g* 25 min, 200,000 *g* 2 h). The exosome pellets were washed with 15 mL PBS and centrifuged at 200,000 *g* for ~2 h to gather the final exosome samples derived from the renal tissues, and then the exosomes were examined by transmission electron microscopy (TEM) and the size distributions of exosomes were clarified by Nanoparticle Tracking Analysis by the Nanosight machine.

For the extraction of the exosomes derived from urine, we gathered 3 mL mice urine samples, and then we conducted this assay given the previously described methods^10^. Specifically, the urine samples were centrifuged at 1500 × *g* for about 20 min to remove cells and residual debris, followed by gradient centrifugation (13,000 *g* 25 min, 200,000 *g* 1 h).

For the extraction of the exosomes derived from renal tubular, we applied a stainless steel sieve (80 mesh) to separate tubular fragments and then purified these exosomes by ultracentrifugation given the previously described methods^11^.

### Quantitative real-time PCR (qRT-PCR)

Given the previously described methods with minor modifications^12^, the qRT-PCR was conducted. The total RNAs were isolated from the mouse kidney tissues, renal TECs TCMK-1, primary mouse renal TECs and the exosomes derived from different sources (kidney, urine, and renal tubular), urine from diabetic patients, and then the high-quality RNAs were transcribed into cDNA using SuperScript™ Double-Stranded cDNA Synthesis Kit (Thermo Fisher Scientific). Real-time PCR was conducted on the ABI 7300 Real-Time qPCR system using an SYBR Green PCR kit (QIAGEN). U6 was from Ribobio Company (Guangzhou, China), and GAPDH was from Sangon Biotech Company (Shanghai, China). U6 and GAPDH were applied as internal references. A 2^−∆∆CT^ method was applied to quantify the relative expression of different molecules. The sequences of all primers applied for qRT-PCR are exhibited in Table 1.

**Table 1:** The sequences of all primers used in qRT-PCR.

Gene name	Primer sequence (5′–3′)
Hsa-miR-483-5p	Forward: GCGAAGACGGGAGGAAAGA
	Reverse: AGTGCAGGGTCCGAGGTATT
Mmu-miR-483-5p	Forward: CGCGAAGACGGGAGAAGAGA
	Reverse: AGTGCAGGGTCCGAGGTATT
Hsa-Col1a1	Forward: CAGGCTGGTGTGATGGGATT
	Reverse: GGGCCTTGTTCACCTCTCTC
Mmu-Col1a1	Forward: CCCAGTGGCGGTTATGACTT
	Reverse: CTCAAGGTCACGGTCACGAA
Hsa-Col4a1	Forward: TTTTGTGATGCACACCAGCG
	Reverse: AGTAATTGCAGGTCCCACGG
Mmu-Col4a1	Forward: AACAACGTCTGCAACTTCGC
	Reverse: CTTCACAAACCGCACACCTG
Mmu-fibronectin	Forward: CGTGATCATCGATGCCTCCA
	Reverse: AGGGGATCCAGGCTTCTCAT
Hsa-fibronectin	Forward: AGCCTGGGAGCTCTATTCCA
	Reverse: CTTGGTCGTACACCCAGCTT
TIMP2	Forward: ATGCTGGGGTTTCTAGCCAC
	Reverse: TGGCACTTTGTCCCAAAGGT
MAPK1	Forward: CTGTCTTCAGCCCGTCTCAG
	Reverse: TTGAAAGTGCACACTGCTGC

### Cell culture and different treatments

The mouse renal TECs TCMK-1 were from American Type Culture Collection (ATCC, USA) and were placed in DMEM (Gibco) with 10% fetal bovine serum (FBS, Gibco) and 1% penicillin/streptomycin (Gibco) and cultured at 37 °C, 5% CO_2_, and then replaced with fresh medium every 2 days.

The HK-2 cells were from ATCC (USA) and were placed in DMEM (Gibco) with the addition of 10% FBS (Gibco) and 1% penicillin/streptomycin (Gibco) and cultured at 37 °C, 5% CO_2_.

TCMK-1 cells were from ATCC (USA) and were treated with 30 mM HG for 48 h to verify the function of miR-483-5p in vitro, and 5 mM D-glucose treatment was applied as a control group. Besides, to maintain the isotonic state after HG treatment, the cells were additionally treated with 30 mM mannitol for nearly 48 h.

### The isolation and culture of primary mouse renal TECs

Given the previously described method with some modifications^13^, we conducted the isolation of primary mouse renal TECs. Specifically, after the mice were fixed supine and anesthetized, the mouse kidney tissues were isolated and cut into small pieces, and were put in 10 mL digestive buffer for incubation. The cortex was separated from the medulla. Next, the above-isolated cortex was placed in collagenase A (Sigma-Aldrich) and incubated at room temperature with shaking for 30 min, and then renal tubules of different sizes were separated through a cell strainer and placed in DMEM medium (Gibco) with the addition of 10% bovine calf serum, 5 µg/mL insulin, 5 µg/mL transferrin, 5 ng/mL selenium, 40 ng/mL hydrocortisone, and 10^−12^ M tri-iodothyronine for further culture.

### Cell transfection

After culturing TCMK-1, HK-2, and primary mouse renal TECs to a fusion of 70–80%, the synthetic miR-483-5p mimic, si-TIMP2, or si-MAPK1 was transfected into TCMK-1 cells using Lipofectamine 2000 Transfection Reagent (Invitrogen) given the standard procedure of the reagent manufacturer. Also, the synthetic miR-483-5p mimic was transfected into HK-2 cells, and the synthetic si-hnrnpa1 or/and miR-483-5p inhibitor was transfected into primary mouse renal TECs. The sequences of siRNAs are exhibited: si-TIMP2: S:UACUGAAUCCUCUUGAUGGGG; as:CCAUCAAGAGGAUUCAGUAUG. si-MAPK1: S:UUGAGAUUAUCAUAAGCAGAG; as:CUGCUUAUGAUAAUCUCAACA. si-HNRNPA1: S:UCUUUUUCACAGUUAAGUGGG; as:CACUUAACUGUGAAAAAGAUC, and the si-NC was purchased from Santa Curz, Inc.

### Western blot

RIPA buffer (Cell Signaling Technology) was applied to extract total proteins from cells and tissues, and the proteins of different molecular weights were separated by SDS-PAGE. Immediately afterward, the proteins were electrically transferred into the PVDF membrane (Roche), and the membrane was put in 5% skim milk and blocked at room temperature for 1 h, and then incubated with specific primary antibodies, including anti-CD63 (ab134045, Abcam), anti-CD9 (ab92726, Abcam), anti-CoL I (ab34710, Abcam), anti-CoL III (ab184993, Abcam), anti-fibronectin (ab2413, Abcam), anti-β-actin (ab8226, Abcam), anti-α-SMA (ab7817, Abcam), anti-E-cadherin (ab40772, Abcam), anti-Vimentin (ab92547, Abcam), anti-psmad3 (#9520, Cell Signaling Technology),anti-smad3 (ab40854, Abcam), anti-nSnail (ab216347, Abcam), anti-Lamin B1 (ab16048, Abcam), anti-TIMP2 (ab230511, Abcam), anti-ERK1/2 (ab184699, Abcam), anti-pERK1/2 (#4370, Cell Signaling Technology), anti-APN (also named CD13, ab108310, Abcam) and anti-HNRNPA1 (ab177152, Abcam) at 4 °C overnight. The membranes were incubated with the secondary antibody (ab205718, Abcam) at room temperature for 1.5 h. The enhanced chemiluminescence reagents (Millipore Sigma) and Image J were applied to observe and analyze protein bands.

### Immunofluorescence

MiR-483-5p mimic was transfected into HK-2 cells and then treated the cells with 30 mM HG for nearly 48 h. The cells were fixed with 4% paraformaldehyde for 30 min, and then the cells were blocked with 0.1% Triton X-100 (Sigma-Aldrich) for nearly 1 h. Next, the cells were incubated with anti-E-cadherin (#3195, Cell Signaling Technology) and anti-α-SMA (#19245, Cell Signaling Technology) antibodies at 4 °C for about 12 h. The cells were put in a secondary fluorescent antibody (Invitrogen) and then incubated for 1 h at room temperature in a dark environment. The cells were stained with DAPI (Sigma-Aldrich) in a dark environment for nearly 5 min.

### Dual-luciferase reporter gene assay

The dual-luciferase reporter gene was conducted to verify the target genes of the top 10 genes (PTMA, SCRT1, CXXC5, TIMP2, CTDSPL2, NXF1, MAPK1, REM2, STK40, and CAMK1D) that efficiently bound to miR-483-5p. In brief, we constructed a luciferase reporter vector that contained the potential miR-483-5p binding sites of the top 10 target genes 3′UTR, and then we co-transfected the above recombinant vector and miR-483-5p mimic into 293T cells. The luciferase activity was tested by a dual-luciferase reporter assay system (Promega).

### RNA pull-down

The biotin-labeled miR-483-5p probe and streptavidin magnetic beads (Invitrogen) were incubated at room temperature and then incubated with TCMK-1 cell lysate or primary mouse renal TEC lysate. Subsequently, the magnetic beads were washed with lysis buffer, and the TIMP2 and MAPK1 bound to the magnetic beads were analyzed by qRT-PCR or the HNRNPA1 protein bound to the magnetic beads was analyzed by western blot.

### In vivo experiment

The constructions of type 1 and type 2 diabetic mouse models were the same as the above methods (2.1 section). Besides, db/db mice were injected with AAV-miR-483-5p or AAV-control (1 × 10^11^) through the tail vein at the 8th week, and C57BL/6 mice were injected with AAV-miR-483-5p or AAV-control (1 × 10^11^) through the tail vein at 8 weeks after the successful establishment of type 1 diabetic mouse model. Ten mice were randomly assigned to each group. Besides, due to the presence of the GFP gene in the AAV vector, we conducted GFP detection on the paraffin sections of the AAV-miR-483-5p injection group. The kidney tissues of each group of mice were isolated for follow-up research. All animal experiment protocols were approved by the Animal Care and Use Committee of Zhengzhou University.

### Immunohistochemistry

Immunohistochemistry experiments were conducted on paraffin-embedded kidney tissue sections using anti-CoL I, anti-CoL III, and anti-α-SMA antibodies. The kidney tissue sections were dewaxed and dehydrated and then put in citrate buffer at 95 °C for 10–15 min, and then the tissue sections were incubated with anti-CoL I (Abcam), anti-CoL III (Abcam) and anti-α-SMA (Abcam) antibodies at 4 °C for about 12 h. The sections were incubated with the secondary antibody (Abcam). The DAB kit (Sangon Biotech) was applied to visualize the slices and counterstained the slices with hematoxylin (Sangon Biotech).

### Detection of serum creatinine and urinary albumin to creatinine ratio

Given the standard method of the reagent manufacturer, the concentrations of serum creatinine and urinary albumin to creatinine ratio were quantified using Creatinine Serum Detection Kit (StressMarq) and urinary albumin to creatinine ratio assay kit (Biovision).

### RNA immunoprecipitation

The interactions between HNRNPA1 and miR-483-5p, HNRNPA2/B1, and miR-483-5p were assessed using an RNA immunoprecipitation kit (Geneseed Biotech). Specifically, the primary mouse renal TECs were lysed and then incubated with an RIP buffer containing anti-HNRNPA1 (Abcam) and anti-HNRNPA2/B1 (Abcam) conjugated magnetic beads. The expression of miR-483-5p was tested by qRT-PCR.

### The co-localization of HNRNPA1 with miR-483-5p

The digoxin-labeled probe sequence of HNRNPA1 and the biotin-labeled probe sequence of miR-483-5p were applied to assess the co-localization of HNRNPA1 and miR-483-5p in cells. The detailed method of the RNA fluorescence in situ hybridization to assess the co-localization of HNRNPA1 and miR-483-5p in cells was conducted referring to the previously described method with minor modifications^14^.

### Clinical samples

The study was approved by the ethics committee of the First Affiliated Hospital of Zhengzhou University. Written informed consent was obtained from all the patients involved. A total of 20 diabetic patients were contained in this research, including 18 patients with type 2 diabetes and 4 patients with type 1 diabetes and the urine samples of all patients were gathered for follow-up studies.

### Statistical analysis

All data were exhibited as mean ± standard deviation. Student’s *t*-test was applied to assess the differences between the two groups, and one-way ANOVA followed by Tukey’s post-test was applied to assess the differences among more than two groups. Pearson correlation coefficient analysis was applied to assess the correlation between urinary ACR and urinary exosome miR-483-5p. A *P* value of less than 0.05 presented a significant difference.

## Results

### Different expressions of miR-483-5p in kidney tissues and exosomes derived from the renal tissues in type 1 and type 2 diabetic mice

**Fig. 1:**
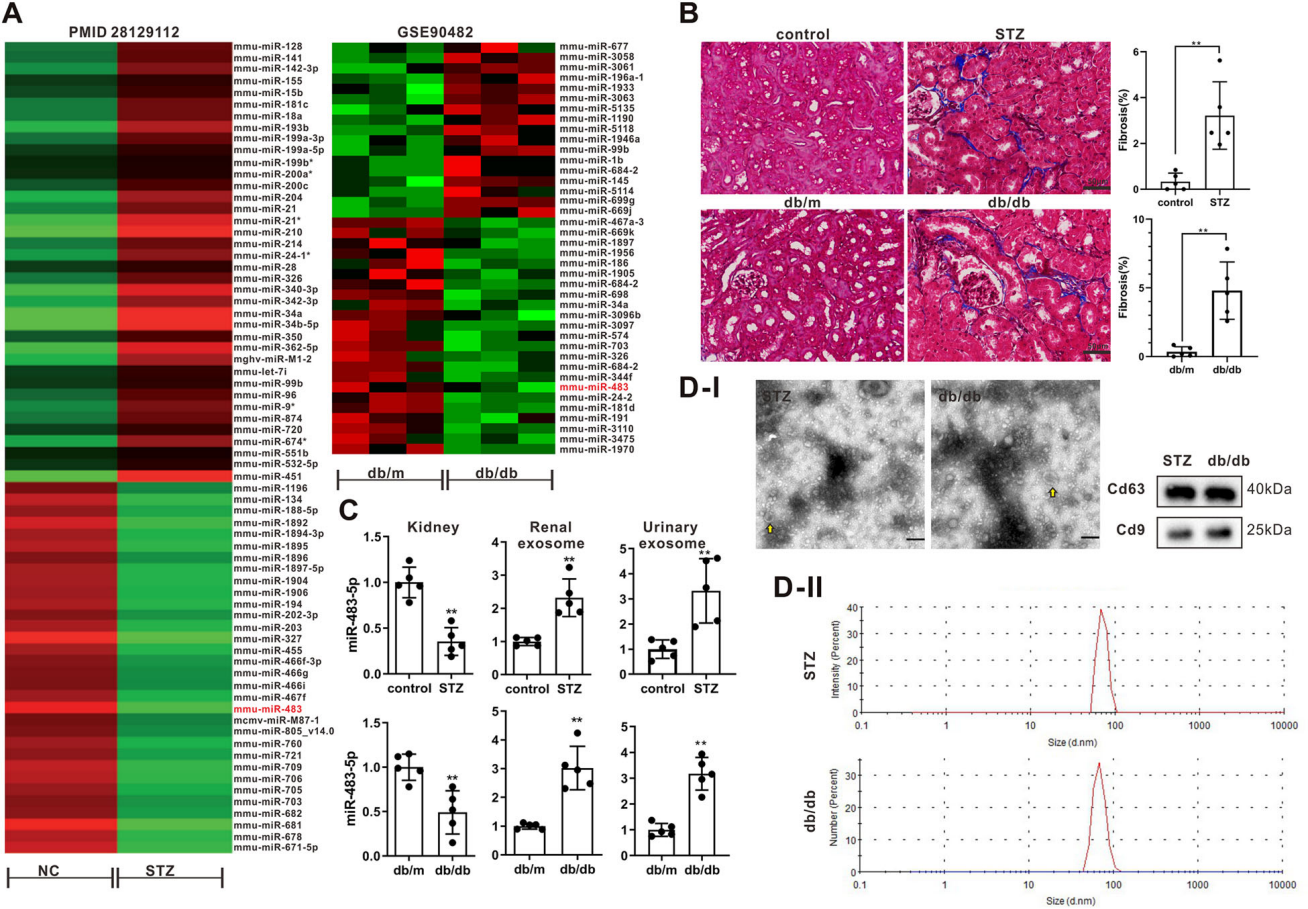
The expressions of miR-483-5p in kidney tissues and exosomes derived from the renal tissues in type 1 and type 2 diabetic mice. **A** PubMed database and GEO datasets (GSE90482) were applied to identify miRNAs that related to diabetic. **B** Masson staining of kidney tissues in type 1 and type 2 diabetic mice (Scale Bar = 50 μm). **C** Detection of the expressions of miR-483-5p in the kidney tissues and exosomes derived from renal tissues and urine in diabetic mice by quantitative real-time PCR (qRT-PCR). **D** Transmission Electron Microscope (TEM) was applied to identify exosomes (arrows pointed to exosomes, Scale Bar = 200 nm), and detection of the protein levels of exosome markers CD63 and CD9 by western blot, and the size distribution of exosomes. **P < 0.01 vs. control or db/m. Student’s *t*-test. STZ Streptozotocin, db/m db/misty.

Through the PubMed database and GEO datasets (GSE90482), miR-483-5p was found to be lowly expressed both in the kidney tissues of type 1 and type 2 diabetic mice (Fig. 1A). Therefore, miR-483-5p was selected for subsequent research. The kidney tissues of type 1 and type 2 diabetic mice were observed by Masson staining, and the results expounded that the kidney tissues of the mice had a typical injury and severe collagen fibrosis, hinting that the diabetic mouse models were successfully constructed (Fig. 1B). Besides, the detection of blood glucose in diabetic mice expounded that compared with the control group, the concentration of blood glucose was raised in diabetic mice, and this proved the successful construction of diabetic mouse models again (Supplementary Fig. 1A). Next, we verified the expression changes of miR-483-5p in diabetic mice, and the results expounded that miR-483-5p expression was lessened in the kidney tissues of diabetic mice, while miR-483-5p expression was raised in the exosomes derived from renal tissues and urine in diabetic mice (Fig. 1C), and these exosomes were identified by TEM (Fig. 1D–I). CD63 and CD9 are the commonly used exosome markers^15^. Western blot analysis expounded that CD63 and CD9 were expressed in the exosomes (Fig. 1D–I). Besides, the size distribution of exosomes was exhibited in Fig. 1D–II, these findings further clarified that the exosomes were successfully isolated. The above results expounded that miR-483-5p expression was lessened in the kidney tissues of diabetic mice, and was raised in the exosomes derived from renal tissues and urine in diabetic mice.

### Verification of miR-483-5p restrains the expressions of fibrosis-related genes in vitro

**Fig. 2:**
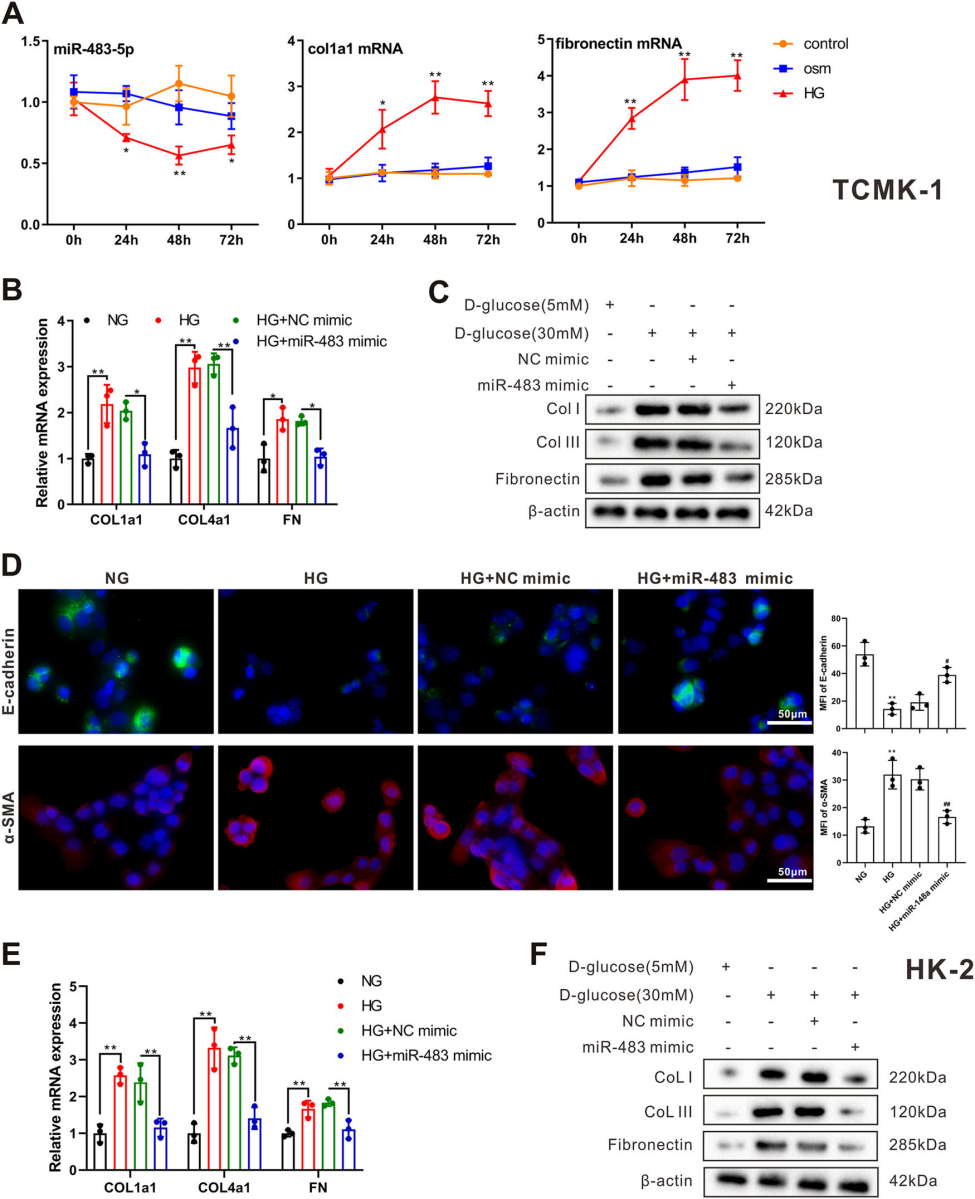
Effect of miR-483-5p on the expressions of fibrosis-related genes in vitro. The in vitro diabetic model was induced by high glucose (HG). **A** Detection of miR-483-5p, Col1a1, and fibronectin expressions by qRT-PCR at 0, 24, 48, and 72 h after the HG treatment. After transfecting miR-483-5p mimic into renal tubular epithelial cells (TECs) TCMK-1, the cells were treated with 30 mM HG for 48 h. **B** Detection of Col1a1, Col4a1, and fibronectin mRNA levels by qRT-PCR. **C** Detection of CoL I, CoL III, and fibronectin protein levels by western blot. After transfecting miR-483-5p mimic into HK-2 cells, the cells were treated with 30 mM HG for 48 h. **D** Detection of E-cadherin and alpha-smooth muscle actin (α-SMA) by immunofluorescence. **E** Detection of Col1a1, Col4a1, and fibronectin mRNA levels by qRT-PCR. **F** Detection of CoL I, CoL III, and fibronectin protein levels by western blot. *P < 0.05, **P < 0.01 vs. control, NG or HG + NC mimic. ^#^*P* < 0.05, ^##^*P* < 0.01 vs. HG + NC mimic. One-way ANOVA followed by Tukey’s post-test for multiple comparisons was applied for groups of three or more of three independent experiments. osm osmolarity, HG high glucose, NG normal glucose, FN fibronectin.

Next, we further probed into the function of miR-483-5p in an in vitro diabetic model induced by HG. Col1a1 and fibronectin are common fibrosis-related molecules and can be applied as fibrosis markers^16,17^. The results of qRT-PCR expounded that miR-483-5p expression was lessened in the HG group, and Col1a1 and fibronectin were raised (Fig. 2A). After transfecting miR-483-5p mimic into mouse-derived renal TECs TCMK-1, the cells were treated with 30 mM HG for 48 h. As exhibited in Fig. 2B, HG treatment raised the mRNA levels of Col1a1, Col4a1, and fibronectin, while these trends were reversed after the transfection of miR-483-5p mimic. Besides, HG treatment raised the protein levels of CoL I, CoL III, and fibronectin, while these trends were reversed after the transfection of miR-483-5p mimic (Fig. 2C). Furthermore, miR-483-5p mimic was transfected into HK-2 cells, and then the cells were treated with 30 mM HG for 48 h. As exhibited in Fig. 2D, the transfection of miR-483-5p mimic restrained fibrosis-related protein alpha-smooth muscle actin (α-SMA) and boosted epithelial cell marker E-cadherin, hinting that the fibrosis and EMT might be restrained after the transfection of miR-483-5p mimic. Besides, the transfection of miR-483-5p mimic lessened the mRNA levels of Col1a1, Col4a1 and fibronectin and the protein levels of CoL I, CoL III, and fibronectin, which expounded that the generation of ECM was restrained after the transfection of miR-483-5p mimic (Fig. 2E, F). These findings expounded that the transfection of miR-483-5p mimic lessened the expressions of fibrosis-related genes in vitro.

### TIMP2 and MAPK1 participate in regulating the effect of miR-483-5p on renal TECs under HG conditions

**Fig. 3:**
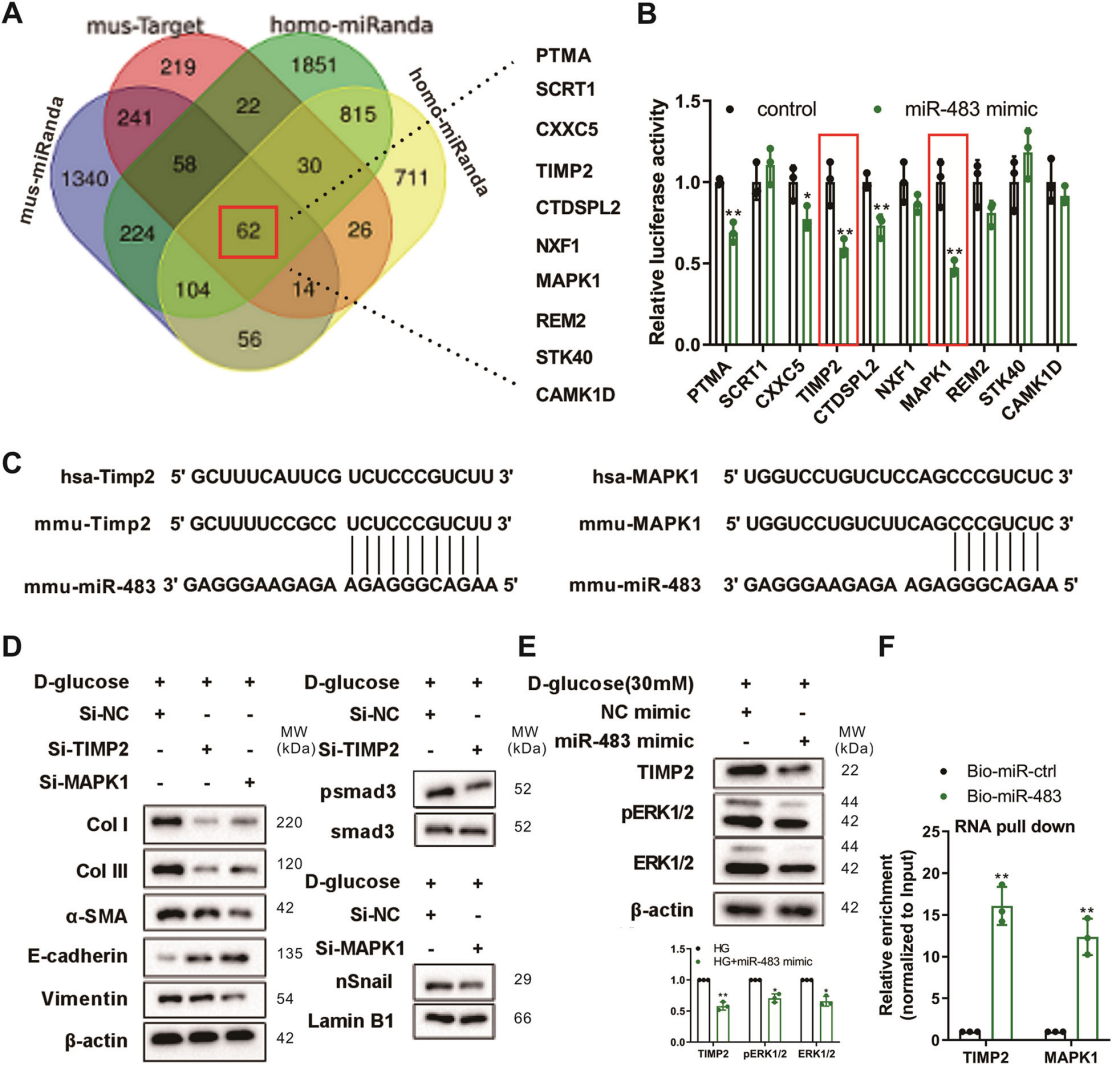
Effect of TIMP2 and MAPK1 on the regulation of miR-483-5p on renal TECs under HG conditions. **A** TargetScan and miRanda databases were applied to predict target genes that were efficiently bound to miR-483-5p both in mice and humans. **B** The top 10 genes (PTMA, SCRT1, CXXC5, TIMP2, CTDSPL2, NXF1, MAPK1, REM2, STK40, and CAMK1D) with strong conservative software prediction scores were applied to conduct the dual-luciferase reporter gene assay. **C** The binding sites of miR-483-5p and TIMP2, miR-483-5p and MAPK1 in mice and humans. **D** After transfecting si-TIMP2, si-MAPK1 into TCMK-1 cells, the cells were treated with 30 mM HG for 48 h. Detection of CoL I, CoL III, α-SMA, E-cadherin, Vimentin, Smad3, and nSnail protein levels by western blot. **E** After transfecting miR-483-5p mimic into TCMK-1 cells, the cells were treated with HG. Detection of TIMP2, ERK1/2 (MAPK1 encoding protein), and pERK1/2 protein levels by western blot. **F** RNA pull-down experiments corroborated that miR-483-5p bound to the 3′UTR region of TIMP2 and MAPK1, respectively. *P < 0.05 vs. HG or control. **P < 0.01 vs. HG, control or Bio-miR-ctrl. Student’s *t*-test. of three independent experiments. nSnail Snail in the nucleus, NC negative control, HG high glucose, ctrl control.

Immediately after, we predicted the target genes that were efficiently bound to miR-483-5p through public databases TargetScan and miRanda. Among the 62 target genes jointly predicted by mice and humans (Fig. 3A), we selected the top 10 genes with strong conservative software prediction scores to conduct dual-luciferase experiments, and we screened out that TIMP2 and MAPK1 had strong binding abilities (Fig. 3B), and their binding sites were conserved between mice and humans (Fig. 3C). After screening the target genes TIMP2 and MAPK1 that efficiently bound to miR-483-5p, we probed into whether TIMP2 and MAPK1 influenced the regulation of miR-483-5p on renal TECs in diabetes under HG conditions. After transfecting si-TIMP2, si-MAPK1 into TCMK-1 cells, the cells were treated with 30 mM HG for 48 h. From the analysis of western blot, the interference with TIMP2 or MAPK1 lessened CoL I, CoL III, α-SMA and Vimentin protein levels, and raised E-cadherin, hinting that the interference with TIMP2 or MAPK1 restrained the generation of ECM and the EMT (Fig. 3D). Also, the interference with TIMP2 restrained the TGF-beta1/pSmad3 pathway, and the interference with MAPK1 lessened the expression of Snail (the key transcription molecule of EMT) in the nucleus, hinting that MAPK1 might regulate EMT by influencing the expression of Snail (Fig. 3D). miR-483-5p mimic was transfected into TCMK-1 cells, and then the cells were treated with HG. As exhibited in Fig. 3E, the overexpression of miR-483-5p lessened TIMP2 and the MAPK1 encoding protein ERK1/2 expressions. Besides, RNA pull-down experiments corroborated that miR-483-5p bound to the 3′UTR region of TIMP2 and MAPK1, respectively (Fig. 3F). Overall, our data corroborated that TIMP2 and MAPK1 were bound up with the regulation of the effect of miR-483-5p on renal TECs under HG conditions.

### Overexpression of miR-483-5p restrains renal interstitial fibrosis in diabetic mice

**Fig. 4:**
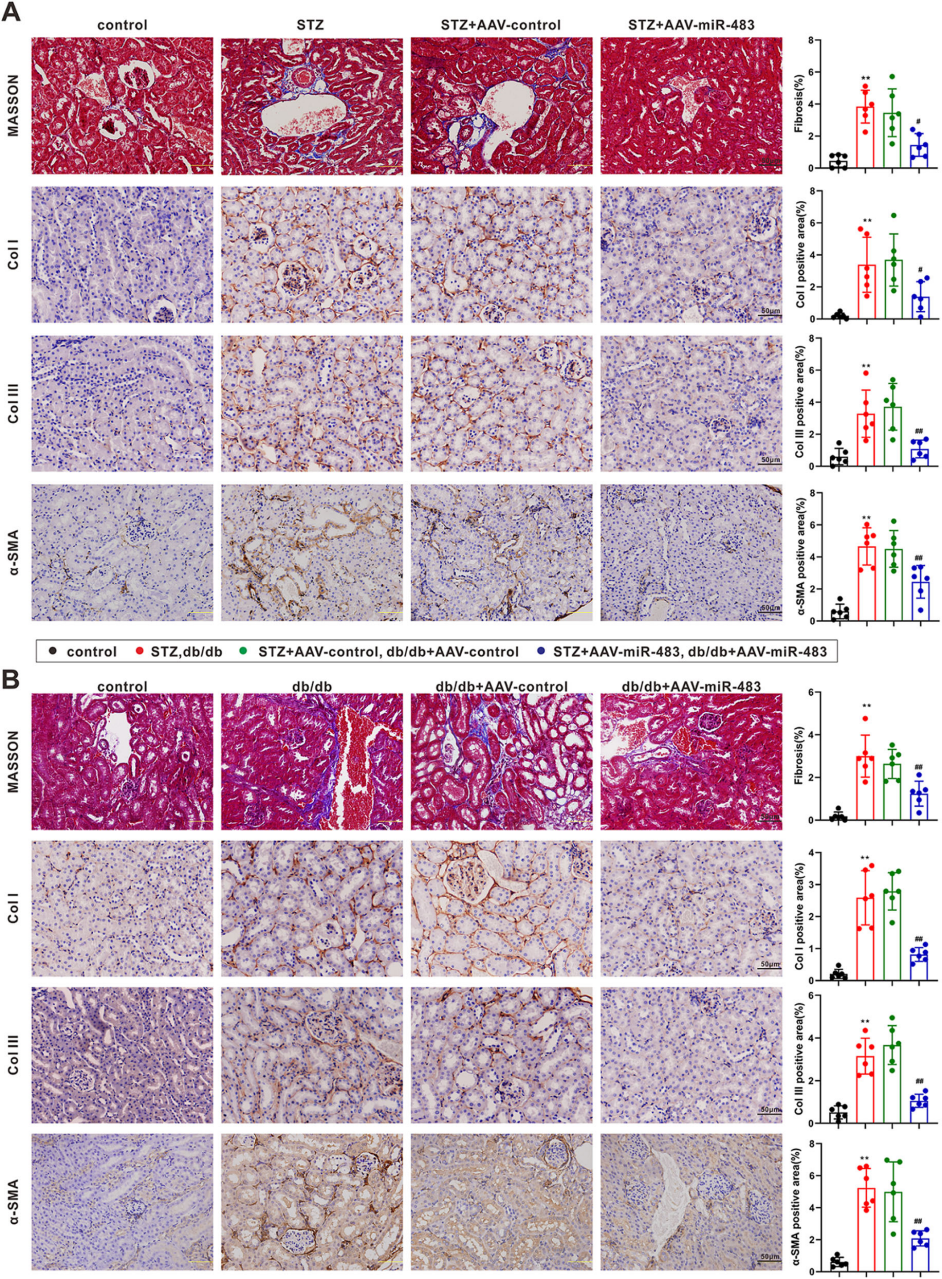
Effect of miR-483-5p on the renal interstitial fibrosis in type 1 and type 2 diabetic mice. **A**, **B** The interstitial fibrosis in diabetic mice was observed by Masson staining and immunohistochemistry (Scale Bar =  50 μm). **P < 0.01 vs. control. ^#^*P* < 0.05 vs. STZ + AAV-control or db/db + AAV-control. One-way ANOVA followed by Tukey’s or LSD post-test. st. STZ Streptozotocin, db/m db/misty, ACR albumin to creatinine ratio.

**Fig. 5:**
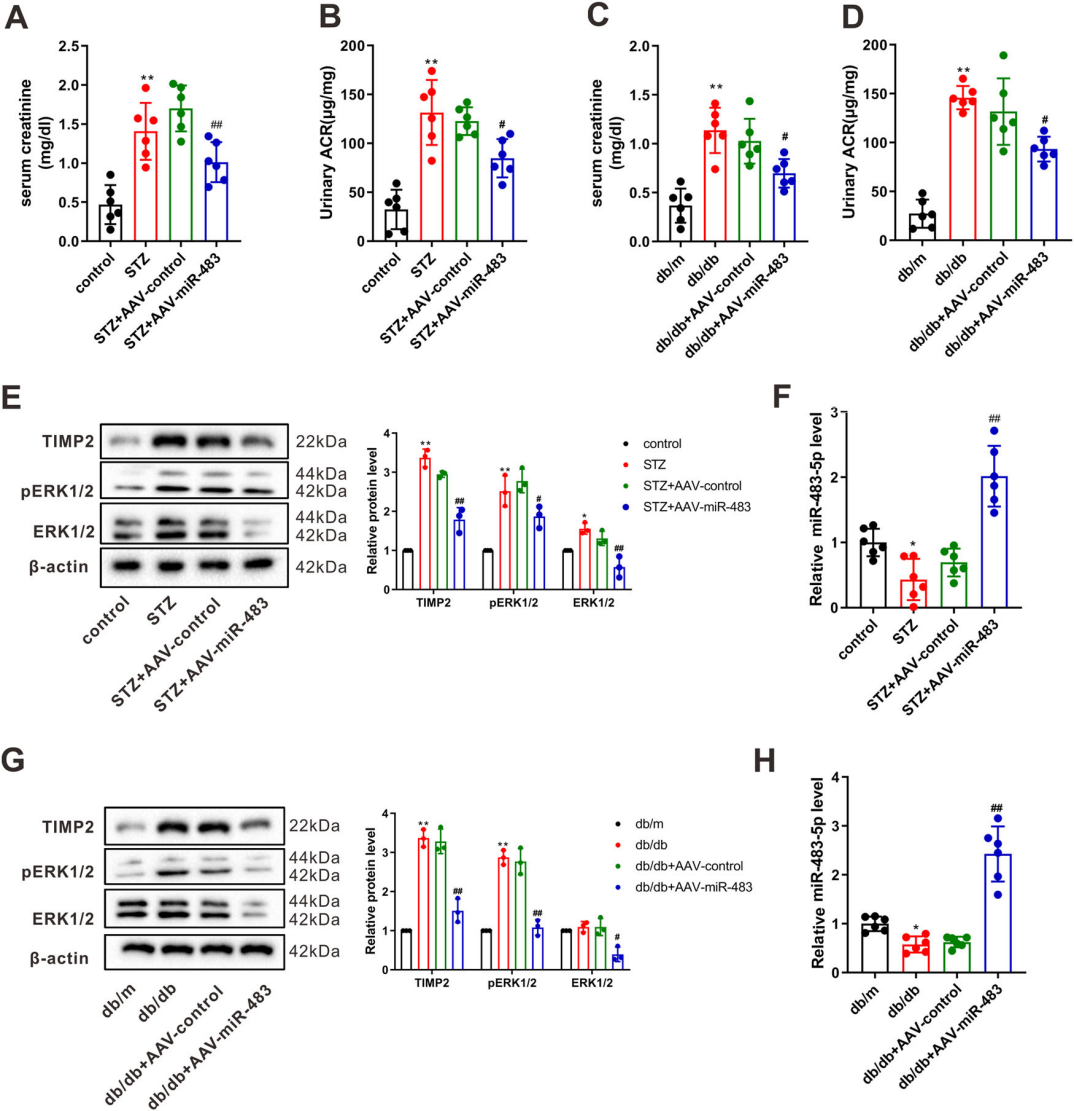
Regulation of miR-483-5p on the renal interstitial fibrosis in type 1 and type 2 diabetic mice. **A**–**D** Detection of serum creatinine and urinary albumin to creatinine ratio concentrations by Creatinine Serum Detection Kit and urinary albumin to creatinine ratio assay kit. **E**–**H** Detection of TIMP2, ERK1/2, and miR-483-5p expressions by qRT-PCR and western blot. *P < 0.05, **P < 0.01 vs. control or db/m. ^#^*P* < 0.05, ^##^*P* < 0.01 vs. STZ + AAV-control or db/db + AAV-control. One-way ANOVA followed by Tukey’s or LSD post-test. STZ Streptozotocin, db/m db/misty, ACR albumin to creatinine ratio.

Furthermore, we probed into the function of miR-483-5p in an in vivo diabetic mice model. From the results of Masson staining and immunohistochemistry, the injection of AAV-miR-483-5p relieved the interstitial fibrosis in diabetic mice (Fig. 4A, B). Serum creatinine and urinary albumin to creatinine ratio are routine indicators of kidney pathological changes in type 1 and type 2 diabetes^18,19^. As exhibited in Fig. 5A–D, the injection of AAV-miR-483-5p ameliorated the function of the kidney in diabetic mice. Moreover, we measured TIMP2, ERK1/2, and miR-483-5p expressions in mouse kidney tissues and clarified that the TIMP2 and ERK1/2 expressions were lessened after the injection of AAV-miR-483-5p, and miR-483-5p was raised (Fig. 5E–H). In summary, we verified that the overexpression of miR-483-5p restrained renal interstitial fibrosis in diabetic mice and negatively regulated the expressions of TIMP2 and ERK1/2.

### miR-483-5p in the mouse model of diabetes is mainly in the exosomes derived from renal tubular

**Fig. 6:**
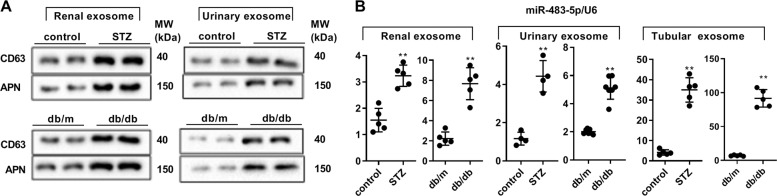
Different expressions of miR-483-5p in the mouse model of diabetes in the exosomes derived from renal tissues, urine, and renal tubular. **A** Detection of CD63 and APN protein levels by western blot. **B** Detection of miR-483-5p expression by qRT-PCR. **P < 0.01 vs. control or db/m. Student’s *t*-test. of three independent experiments. STZ Streptozotocin, db/m db/misty, APN Aminopeptidase N.

After constructing the diabetic mouse model, we gathered the exosomes derived from renal tissues, urine and renal tubular, respectively. Aminopeptidase N (APN), also named CD13, is an important marker of exosomes from the renal tubular^20–22^. As exhibited in Fig. 6A, the CD63 and APN in the exosomes derived from renal tissues and urine in type 1 and type 2 diabetic mice were higher than that in the control group, hinting that the above exosomes were successfully isolated and the expression of APN was more obvious in exosomes derived from renal tissues. Moreover, miR-483-5p expression was raised in the exosomes derived from renal tissues, urine and renal tubular in type 1 and type 2 diabetic mice, especially in the exosomes derived from renal tubular (Fig. 6B). The above data expounded that miR-483-5p in diabetic mice was mainly in the exosomes derived from renal tubular.

### The transport protein HNRNPA1-mediated exosomal sorting transported cellular miR-483-5p out of TECs into the urine

**Fig. 7:**
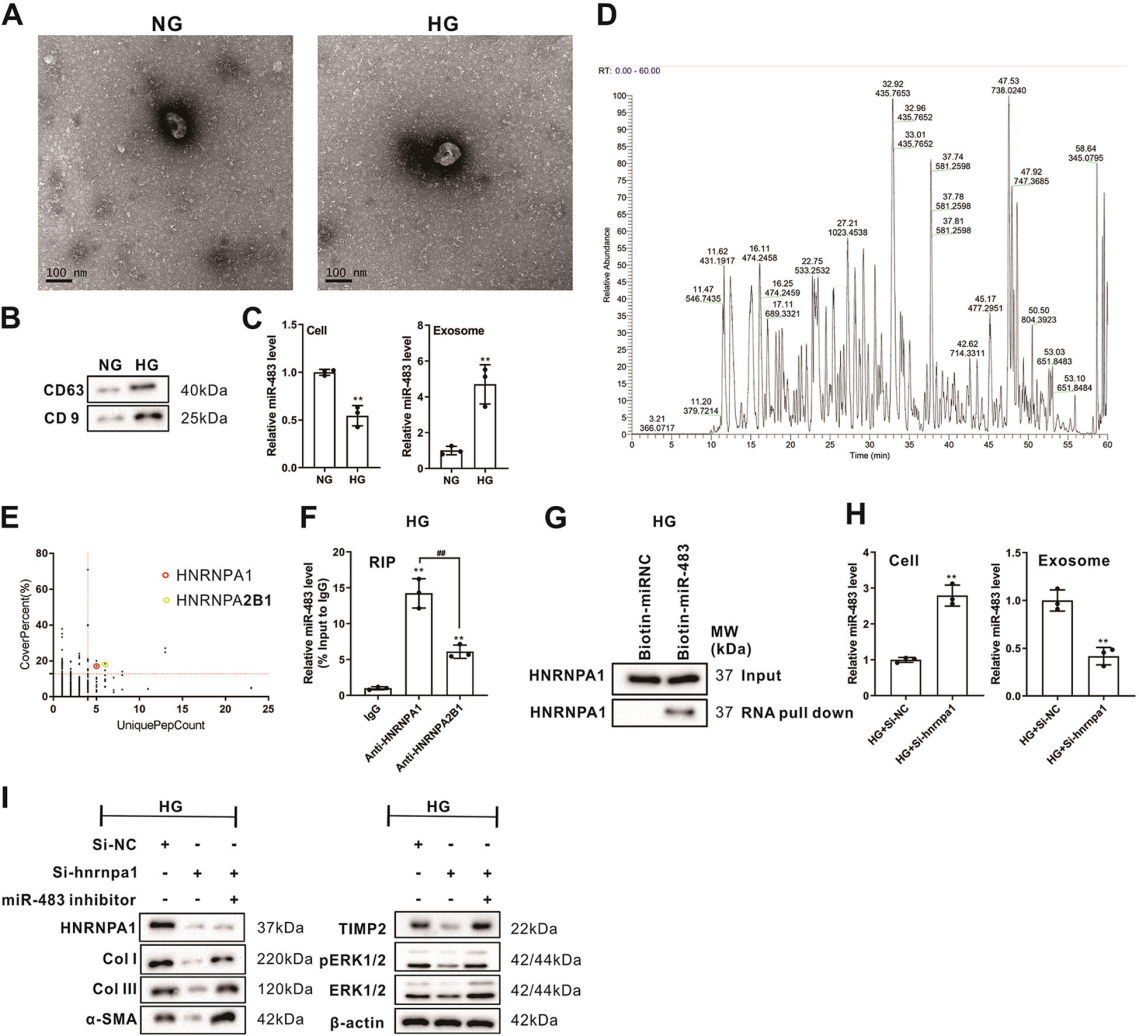
Effect of the transport protein HNRNPA1 on the exosomal sorting of miR-483-5p in renal TECs. **A** The primary mouse renal TECs were cultured under NG and HG conditions for 48 h, and their exosomes were isolated for verification by TEM (Scale Bar = 100 nm) and **B** western blot (CD63 and CD9 proteins). **C** Detection of the miR-483-5p levels in cells and exosomes by qRT-PCR. **D** After HG treatment for 48 h, the RNA pull-down of miR-483-5p was carried out, and the eluate was subjected to mass spectrometry. **E** Gene Ontology (GO) analysis. **F** RNA immunoprecipitation verification of HNRNPA1 and HNRNPA2/B1 that bound to miR-483-5p. **G** RNA pull-down experiment corroborated the binding of HNRNPA1 to miR-483-5p. **H** After transfecting si-hnrnpa1 into primary mouse renal TECs, the cells were treated with 30 mM HG for 48 h. Detection of the miR-483-5p levels in cells and exosomes by qRT-PCR. **I** After transfecting si-hnrnpa1 and/or miR-483-5p inhibitor into primary mouse renal TECs, the cells were treated with 30 mM HG for 48 h. Detection of HNRNPA1, CoL I, CoL III, TIMP2, ERK1/2, and pERK1/2 protein levels by western blot. **P < 0.01 vs. NG, IgG or HG + si-NC. ^##^*P* < 0.01 vs. Anti-HNRNPA1. Student’s *t*-test and one-way ANOVA followed by Tukey’s post-test. of three independent experiments. NG normal glucose, HG high glucose, NC negative control.

Furthermore, we probed into the potential mechanism of the exosomal sorting of miR-483-5p in renal TECs. First, the primary mouse renal TECs were isolated and the identification of renal TECs was exhibited in Supplementary Fig. 1G, and were cultured under NG and HG conditions for nearly 48 h, and their exosomes were isolated and verified by TEM and western blot. As exhibited in Fig. 7A, B, the exosomes were successfully isolated. After HG treatment, the expression of miR-483-5p was lessened in cells, while miR-483-5p was raised in exosomes (Fig. 7C). After HG treatment, the RNA pull-down of miR-483-5p was carried out, and the eluate was subjected to mass spectrometry, and a total of 2101 proteins were found (Fig. 7D). Next, 7 proteins were found through the comprehensive analysis of Uniquepercount and Coverpercent, and then combined with Gene Ontology (GO) analysis, HNRNPA1 (marked with a red circle) and HNRNPA2/B1 (marked with a yellow circle) were found to be bound up with the process of miRNAs sorting to exosomes (Fig. 7E), and this was also consistent with the previous conclusions^23,24^. Therefore, we focused on RIP verification of these two proteins and corroborated that HNRNPA1 was more bound to miR-483-5p (Fig. 7F), and as reported, HNRNPA1 boosts collagen synthesis^25^, so we chose HNRNPA1 for follow-up research. As exhibited in Fig. 7G, the RNA pull-down experiment further corroborated the binding of HNRNPA1 to miR-483-5p again. Furthermore, the RIP and RNA pull-down were also be carried out in NG group, and the RIP assay corroborated that HNRNPA1 bound to miR-483-5p under NG conditions, while the RNA pull-down did not corroborate that the binding of HNRNPA1 to miR-483-5p (Supplementary Fig. 1C, D). The comparison of RIP under HG and NG conditions expounded that the binding efficiency of HNRNPA1 and miR-483-5p was higher under HG conditions was higher than that of NG (Supplementary Fig. 1E). Besides, the detection of the co-localization of HNRNPA1 with miR-483-5p and corroborated that the localization of HNRNPA1 and miR-483-5p were co-located in the cytoplasm (Supplementary Fig. 1F). After si-hnrnpa1 was transfected into primary mouse renal TECs, the cells were treated with HG, and we corroborated that miR-483-5p expression was raised in the cells, while miR-483-5p was lessened in exosomes (Fig. 7H) and contributed to the generation of ECM (Fig. 7I), while the transfection of miR-483-5p inhibitor reversed the regulation of ECM by si-hnrnpa1 (Fig. 7I). The above experimental results corroborated that the transport protein HNRNPA1-mediated exosomal sorting transported cellular miR-483-5p out of TECs, and further influenced the regulation of miR-483-5p on target molecules by regulating the intracellular miR-483-5p expression.

## Discussion

**Fig. 8:**
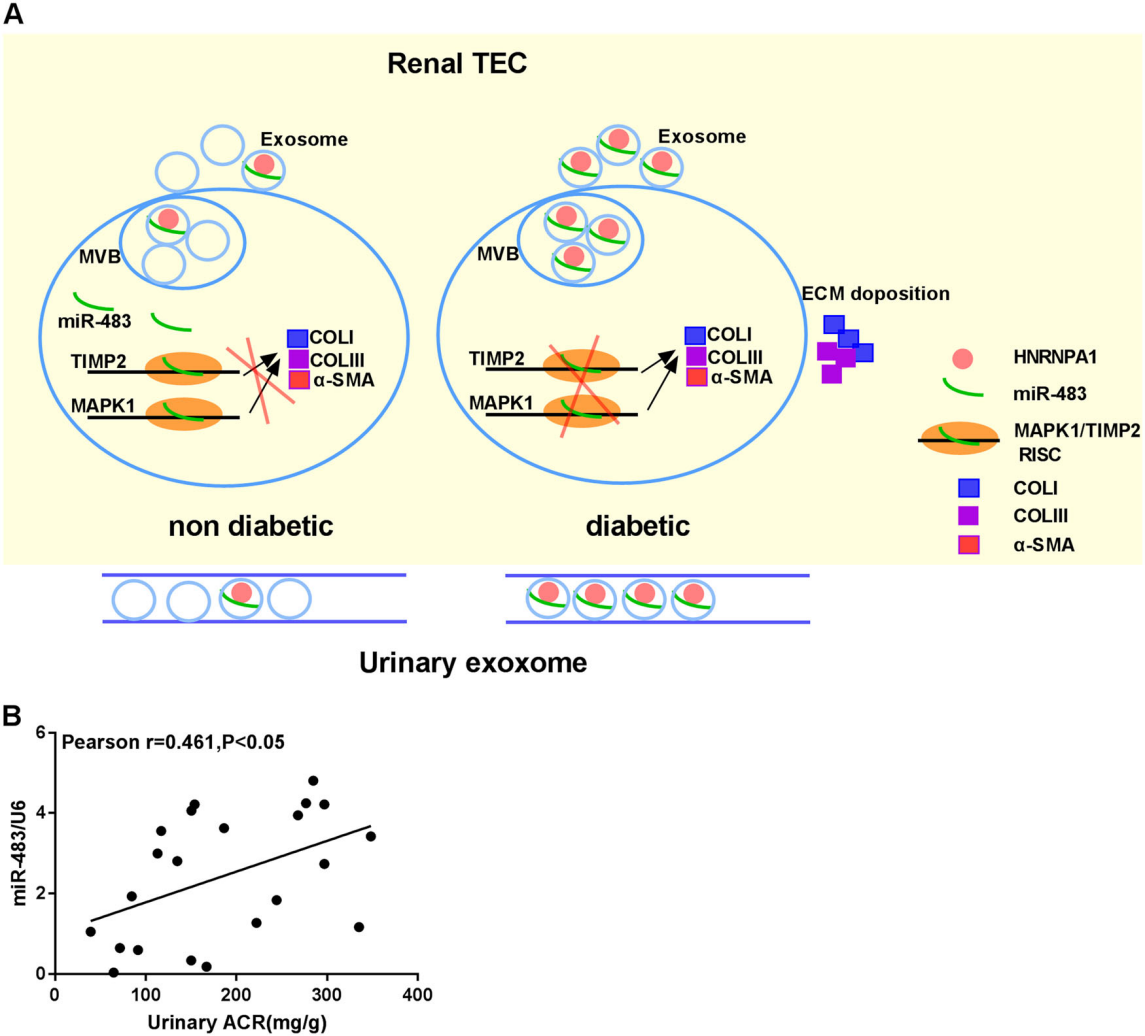
Transport mechanism. **A** Mechanism flow diagram. Under physiological conditions (non diabetic), most miR-483 (miR-483-5p) remained in the renal tubular epithelial cells, and only a small amount of miR-483 was encapsulated into MVB (multivesicular body, vesicles) and was secreted into the extracellular through exosomes. Therefore, the intracellular miR-483 blocked ECM synthesis by restraining the TIMP2/MAPK1 pathway; Under pathological conditions, most miR-483 was encapsulated into MVB and was secreted into the extracellular domain through exosomes and transferred into the urine. Therefore, miR-483 was highly expressed in the urinary exosomes, and the lack of miR-483 in renal tubular epithelial cells led to the activation of the TIMP2/MAPK1 pathway, thus promoting ECM synthesis. **B** Pearson correlation coefficient analysis of the correlation between urinary ACR and urinary exosome miR-483-5p. Pearson correlation coefficient analysis of three independent experiments.

This study mainly probed into the role and mechanism of miR-483-5p in interstitial fibrosis in DN for the first time and corroborated that miR-483-5p expression was lessened in type 1 and type 2 diabetic mouse model and HG-induced renal TEC model. Based on this finding, our further studies corroborated that under the pathological conditions of diabetes, HNRNPA1-mediated exosomal sorting transported cellular miR-483-5p out of TECs into the urine, thus lessening the restraint of cellular miR-483-5p on MAPK1 and TIMP2 mRNAs, and ultimately boosting ECM deposition and the progression of DN-induced renal interstitial fibrosis. The main mechanism diagram of this study is exhibited in Fig. 8A. Importantly, urinary ACR in urine samples of diabetic patients was positively correlated with the expression of miR-483-5p in the exosomes derived from urine (Fig. 8B), hinting that miR-483-5p might also be bound up with the process of renal pathology in clinical practice.

Several previous studies confirm that miRNAs are bound up with the regulation of the progression of DN-induced renal interstitial fibrosis through binding to their target genes^26,27^. As reported, miR-342 blocks renal interstitial fibrosis in DN by lessening SOX6^28^. The targeted restraint of miR-21 alleviates DN by boosting target smad7 expression to restrain TGF-β1-mediated fibrosis in DN^29^. Here, we aimed to probe into the molecules that played functions both in type 1 and type 2 diabetes and discovered that miR-483-5p was lowly expressed in both type I and type II diabetic mice. Therefore, we chose miR-483-5p for further research. For the further mechanism studies, the target genes that efficiently bound to miR-483-5p were TIMP2 and MAPK1, and this conclusion was also corroborated by an RNA pull-down assay, hinting that miR-483-5p might participate in the regulation of type 1 and type 2 diabetes through TIMP2 and MAPK1.

TIMP2 is the only TIMP protein that has both MMP inhibitor and activator functions^30–32^. TIMP2 boosts the activation of MMP2 in cells after renal injury and is reported in the response of fibroblasts and myocardial injury^33,34^. Although MMP2 has the function of degrading ECM, it has been corroborated that is a molecule that boosts fibrosis and EMT^35^. Besides, studies expound that MMP boosts the TGF-beta1 signaling pathway and EMT or collagen synthesis^36–39^. Here, we also corroborated that under HG conditions, the interference with TIMP2 restrained ECM production, EMT and the TGF-beta1/pSmad3 signaling pathway, and TIMP2 expression was negatively regulated by miR-483-5p, hinting that TIMP2 was bound up with the regulation of miR-483-5p on renal TECs under HG conditions. MAPK1, which encodes the ERK2 protein, participates in the MAPK/ERK signaling pathway, and the ERK signaling pathway is bound up with the process of renal fibrosis caused by hypoxia^40^. From the mechanism analysis, the MAPK signaling pathway is bound up with the EMT induced by TGF-beta1^41^. Besides, ERK2 boosts the translocation of Snail1 (an important transcription factor for EMT) of cancer-related fibroblasts into the nucleus^42^. In our present study, under HG conditions, the interference with MAPK1 restrained ECM production and EMT, and lessened Snail in the nucleus, hinting that MAPK1 might affect the key EMT related molecule Snail and thus regulated EMT. Besides, MAPK1 expression was negatively regulated by miR-483-5p, hinting that MAPK1 was also bound up with the regulation of miR-483-5p on renal TECs under HG conditions.

Increasing public data show that exosomes, as a kind of extracellular vesicles (EVs), play important functions in cell communication of different biological processes^43^. Urinary exosomes are generally secreted by podocytes, proximal tubules, distal tubules and other epithelial cells^44^, and studies have expounded that the changes in miRNAs expressions in urinary exosomes characterize the progression of DN^45,46^. In our preliminary experiments, miR-483-5p expression was raised in urinary exosomes of diabetic mice, while was lessened in kidney tissues (Fig. 1C), hinting that miR-483-5p might characterize the progression of DN. Besides, Sonoda et al. proposed that the changes in miRNAs expressions in exosomes largely depend on the intracellular transport and sorting of exosomes^47^, and the study of Li et al. expounded that let-7c-5p expression is raised in the exosomes of DN patients, and is bound up with renal function and DN progression^48^. Therefore, we speculated that the differences in expression of miR-483-5p in TECs between intracellular and extracellular might be due to exosomes mediating the transport of miR-483-5p. In the current research, we corroborated that the highly expressed miR-483-5p in the exosomes derived from renal tubular, and the transporters HNRNPA1 and miR-483-5p tightly bound to each other and the restraint of HNRNPA1 in HG-treated TECs raised the expression of miR-483-5p in cells and lessened miR-483-5p in exosomes, hinting that the transporter HNRNPA1-mediated exosomal sorting transported cellular miR-483-5p out of TECs into the urine.

Overall, our results corroborated that under the pathological conditions of diabetes, HNRNPA1-mediated exosomal sorting transported cellular miR-483-5p out of TECs into the urine, thus lessening the restraint of cellular miR-483-5p on MAPK1 and TIMP2 mRNAs, and finally boosting ECM deposition and the progression of DN-induced renal interstitial fibrosis. Importantly, Urinary ACR in urine samples of diabetic patients was positively correlated with the expression of miR-483-5p in the exosomes derived from urine, hinting that miR-483-5p might also be bound up with the process of renal pathology in clinical practice. This might provide new insights for the diagnosis and treatment of DN, which was of great significance.

## Supplementary information

Supplementary Figure 1

Supplementary Figure 1

## References

[1] Packham DK (2012). Relative incidence of ESRD versus cardiovascular mortality in proteinuric type 2 diabetes and nephropathy: results from the DIAMETRIC (Diabetes Mellitus Treatment for Renal Insufficiency Consortium) database. Am. J. Kidney Dis..

[2] Loeffler I, Wolf G (2015). Epithelial-to-mesenchymal transition in diabetic nephropathy: fact or fiction?. Cells.

[3] Strutz F, Zeisberg M (2006). Renal fibroblasts and myofibroblasts in chronic kidney disease. JASN.

[4] Mora C, Navarro JF (2004). Inflammation and pathogenesis of diabetic nephropathy. Metab. Clin. Exp..

[5] Correia de Sousa M, Gjorgjieva M, Dolicka D, Sobolewski C, Foti M (2019). Deciphering miRNAs' Action through miRNA Editing. Int. J. Mol. Sci..

[6] Lu TX, Rothenberg ME (2018). MicroRNA. J. Allergy Clin. Immunol..

[7] Cheng, Y., Wang, D., Wang, F., Liu, J. Endogenous miR-204 protects the kidney against chronic injury in hypertension and diabetes. **31**, 1539–1554 (2020). 10.1681/ASN.2019101100 PMC7350998 32487559

[8] Kölling M (2017). Therapeutic miR-21 silencing ameliorates diabetic kidney disease in mice. Mol. Ther. J. Am. Soc. Gene Ther..

[9] Borges FT (2013). TGF-β1-containing exosomes from injured epithelial cells activate fibroblasts to initiate tissue regenerative responses and fibrosis. JASN.

[10] Kurahashi R., Kadomatsu T. MicroRNA-204-5p: a novel candidate urinary biomarker of Xp11.2 translocation renal cell carcinoma. **110**, 1897–1908 (2019) 10.1111/cas.14026 PMC6549932 31006167

[11] Li J, Zelenin S, Aperia A, Aizman O (2006). Low doses of ouabain protect from serum deprivation-triggered apoptosis and stimulate kidney cell proliferation via activation of NF-kappaB. JASN.

[12] Li P (2020). LINC00271 inhibits epithelial-mesenchymal transition of papillary thyroid cancer cells by downregulating trefoil factor 3 expression. Aging Pathobiol. Therapeutics.

[13] Ding W, Yousefi K, Shehadeh LA (2018). Isolation, characterization, and high throughput extracellular flux analysis of mouse primary renal tubular epithelial cells. J. Vis. Exp.

[14] Wu X. S. et al. LncRNA-PAGBC acts as a microRNA sponge and promotes gallbladder tumorigenesis. **18**, 1837–1853 (2017). 10.15252/embr.201744147 PMC5623869 28887321

[15] Khushman M (2017). Exosomal markers (CD63 and CD9) expression pattern using immunohistochemistry in resected malignant and nonmalignant pancreatic specimens. Pancreas.

[16] Marchant V (2015). Tubular overexpression of Gremlin in transgenic mice aggravates renal damage in diabetic nephropathy. Am. J. Physiol. Ren. Physiol..

[17] Seo E, Kang H, Oh YS, Jun HS (2017). Psoralea corylifolia L. Seed extract attenuates diabetic nephropathy by inhibiting renal fibrosis and apoptosis in streptozotocin-induced diabetic mice. Nutrients.

[18] Colombo M (2020). Comparison of serum and urinary biomarker panels with albumin/creatinine ratio in the prediction of renal function decline in type 1 diabetes. Diabetologia.

[19] Friedman AN (2008). Value of urinary albumin-to-creatinine ratio as a predictor of type 2 diabetes in pre-diabetic individuals. Diabetes Care.

[20] Dang VD, Jella KK, Ragheb RRT, Denslow ND, Alli AA (2017). Lipidomic and proteomic analysis of exosomes from mouse cortical collecting duct cells. FASEB J..

[21] Yu Y (2018). Non-proximal renal tubule-derived urinary exosomal miR-200b as a biomarker of renal fibrosis. Nephron.

[22] Stange T, Kettmann U, Holzhausen HJ (2000). Immunoelectron microscopic demonstration of the membrane proteases aminopeptidase N/CD13 and dipeptidyl peptidase IV/CD26 in normal and neoplastic renal parenchymal tissues and cells. EJH.

[23] Gao X (2019). Chronic myelogenous leukemia cells remodel the bone marrow niche via exosome-mediated transfer of miR-320. Theranostics.

[24] Villarroya-Beltri C (2013). Sumoylated hnRNPA2B1 controls the sorting of miRNAs into exosomes through binding to specific motifs. Nat. Commun..

[25] Thiele BJ (2004). RNA-binding proteins heterogeneous nuclear ribonucleoprotein A1, E1, and K are involved in post-transcriptional control of collagen I and III synthesis. Circulation Res..

[26] Conserva, F. et al. Urinary miRNA-27b-3p and miRNA-1228-3p correlate with the progression of kidney fibrosis in diabetic nephropathy. **9**, 11357 (2019). 10.1038/s41598-019-47778-1 PMC6684817 31388051

[27] Cardenas-Gonzalez M (2017). Identification, confirmation, and replication of novel urinary microrna biomarkers in lupus nephritis and diabetic nephropathy. Clin. Chem..

[28] Jiang ZH (2020). miRNA-342 suppresses renal interstitial fibrosis in diabetic nephropathy by targeting SOX6. Int. J. Mol. Med..

[29] Wang JY (2014). miR-21 overexpression enhances TGF-β1-induced epithelial-to-mesenchymal transition by target smad7 and aggravates renal damage in diabetic nephropathy. Mol. Cell. Endocrinol..

[30] Wang Z (2014). TIMP2 and TIMP3 have divergent roles in early renal tubulointerstitial injury. Kidney Int..

[31] Hernandez-Barrantes S (2000). Binding of active (57 kDa) membrane type 1-matrix metalloproteinase (MT1-MMP) to tissue inhibitor of metalloproteinase (TIMP)-2 regulates MT1-MMP processing and pro-MMP-2 activation. J. Biol. Chem..

[32] Strongin AY (1995). Mechanism of cell surface activation of 72-kDa type IV collagenase. Isolation of the activated form of the membrane metalloprotease. J. Biol. Chem..

[33] Kandalam V (2010). TIMP2 deficiency accelerates adverse post-myocardial infarction remodeling because of enhanced MT1-MMP activity despite lack of MMP2 activation. Circulation Res..

[34] Kandalam V (2011). Lack of tissue inhibitor of metalloproteinases 2 leads to exacerbated left ventricular dysfunction and adverse extracellular matrix remodeling in response to biomechanical stress. Circulation.

[35] Koszegi, S., Molnar, A. RAAS inhibitors directly reduce diabetes-induced renal fibrosis via growth factor inhibition. **597**, 193–209 (2019) 10.1113/JP277002 PMC6312411 30324679

[36] Kassiri Z (2009). Simultaneous transforming growth factor beta-tumor necrosis factor activation and cross-talk cause aberrant remodeling response and myocardial fibrosis in Timp3-deficient heart. J. Biol. Chem..

[37] Sato M, Muragaki Y, Saika S, Roberts AB, Ooshima A (2003). Targeted disruption of TGF-beta1/Smad3 signaling protects against renal tubulointerstitial fibrosis induced by unilateral ureteral obstruction. J. Clin. Investig..

[38] Karsdal MA (2002). Matrix metalloproteinase-dependent activation of latent transforming growth factor-beta controls the conversion of osteoblasts into osteocytes by blocking osteoblast apoptosis. J. Biol. Chem..

[39] Einecke G (2010). A molecular classifier for predicting future graft loss in late kidney transplant biopsies. J. Clin. Investig..

[40] Liu M (2017). Signalling pathways involved in hypoxia-induced renal fibrosis. J. Cell. Mol. Med..

[41] Lv ZM (2011). The role of the p38 MAPK signaling pathway in high glucose-induced epithelial-mesenchymal transition of cultured human renal tubular epithelial cells. PloS One.

[42] Zhang, K. et al. Mechanical signals regulate and activate SNAIL1 protein to control the fibrogenic response of cancer-associated fibroblasts. **129**,1989–2002 (2016). 10.1242/jcs.180539 PMC4878991 27076520

[43] Jackson CE, Scruggs BS, Schaffer JE, Hanson PI (2017). Effects of inhibiting VPS4 support a general role for ESCRTs in extracellular vesicle biogenesis. Biophys. J..

[44] Pisitkun T, Shen RF, Knepper MA (2004). Identification and proteomic profiling of exosomes in human urine. Proc. Natl Acad. Sci. USA.

[45] Xie, Y., Jia, Y., Cuihua, X., Hu, F. Urinary exosomal microRNA profiling in incipient type 2 diabetic kidney disease. **2017**, 6978984 (2017). 10.1155/2017/6978984 PMC5605810 29038788

[46] Eissa S, Matboli M, Aboushahba R, Bekhet MM, Soliman Y (2016). Urinary exosomal microRNA panel unravels novel biomarkers for diagnosis of type 2 diabetic kidney disease. J. Diabetes Complicat..

[47] Sonoda H (2019). miRNA profiling of urinary exosomes to assess the progression of acute kidney injury. Sci. Rep..

[48] Li W, Yang S, Qiao R, Zhang J (2018). Potential value of urinary exosome-derived let-7c-5p in the diagnosis and progression of type II diabetic nephropathy. Clin. Lab..

