# Supplementary material for: HNRNPA1-mediated exosomal sorting of miR-483-5p out of renal tubular epithelial cells promotes the progression of diabetic nephropathy-induced renal interstitial fibrosis
Source: Cell Death Dis. 2021 Mar 10;12(3):255. doi: 10.1038/s41419-021-03460-x (PMC7946926; doi:10.1038/s41419-021-03460-x)
Supplement: Supplementary file 1 — Supplementary Figure 1 [file 41419_2021_3460_MOESM1_ESM.docx]

**Supplementary Figure 1**

(A) The detection of blood glucose was conducted in diabetic mice at week 4, 8, 12, 16 and 20. (B) The GFP detection was conducted on the paraffin sections of the AAV-miR-483-5p injection group (Scale Bar = 50 μm). (C) RNA immunoprecipitation and (D) RNA pull-down assays were carried out in NG group to clarify whether HNRNPA1-mediated sorting of miR-483-5p was restricted in the pathological condition. (E) The comparison of RIP under HG and NG conditions. (F) The detection of the co-localization of HNRNPA1 with miR-483-5p *in vitro* (scale bar: 20 μm). (G) The identification of renal TECs (×1000). ***P*<0.01 vs. control, IgG or NG. Student's *t*-test. of three independent experiments. NG: normal glucose, HG: high glucose, STZ: Streptozotocin.
