# Supplementary figures and images for: HNRNPA1-mediated exosomal sorting of miR-483-5p out of renal tubular epithelial cells promotes the progression of diabetic nephropathy-induced renal interstitial fibrosis
Source: Cell Death Dis. 2021 Mar 10;12(3):255. doi: 10.1038/s41419-021-03460-x (PMC7946926; doi:10.1038/s41419-021-03460-x)

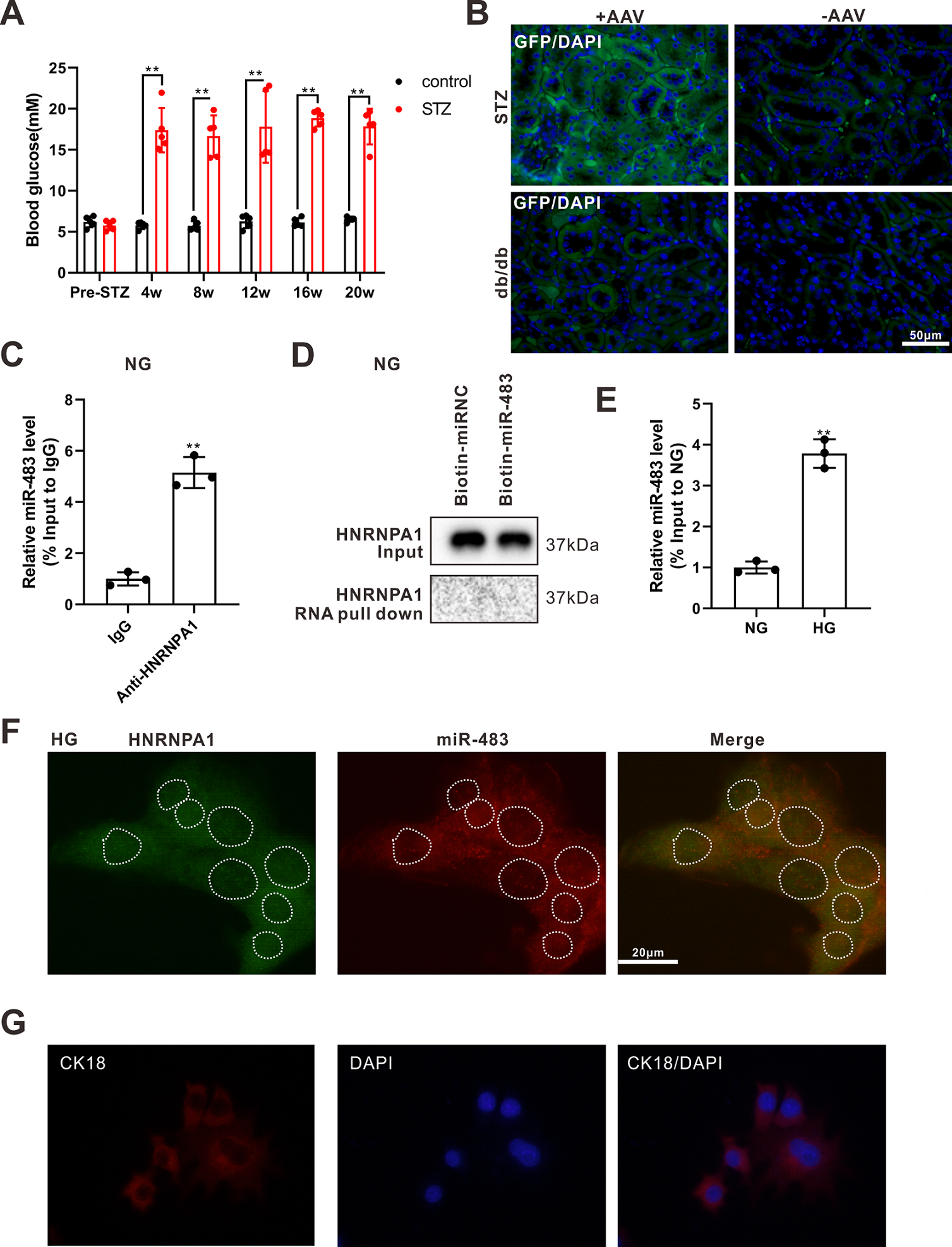

Supplement: Supplementary file 2 — Supplementary Figure 1 [file 41419_2021_3460_MOESM2_ESM.tif]
